# Crystal Structure of Botulinum Neurotoxin A2 in Complex with the Human Protein Receptor SV2C Reveals Plasticity in Receptor Binding

**DOI:** 10.3390/toxins10040153

**Published:** 2018-04-12

**Authors:** Robert Gustafsson, Sicai Zhang, Geoffrey Masuyer, Min Dong, Pål Stenmark

**Affiliations:** 1 Department of Biochemistry and Biophysics, Stockholm University, S-106 91 Stockholm, Sweden; robert.gustafsson@dbb.su.se (R.G.); geoffrey.masuyer@dbb.su.se (G.M.); 2 Department of Urology, Boston Children’s Hospital, Department of Microbiology and Immunobiology and Department of Surgery, Harvard Medical School, Boston, MA 02115, USA; sicai.zhang@childrens.harvard.edu

**Keywords:** botulinum toxin, cell surface receptor, N-linked glycosylation, neurotoxin, protein structure, protein–protein interaction, extracellular domain, membrane protein, structural biology, X-ray crystallography

## Abstract

Botulinum neurotoxins (BoNTs) are a family of highly dangerous bacterial toxins, with seven major serotypes (BoNT/A-G). Members of BoNTs, BoNT/A1 and BoNT/B1, have been utilized to treat an increasing number of medical conditions. The clinical trials are ongoing for BoNT/A2, another subtype of BoNT/A, which showed promising therapeutic properties. Both BoNT/A1 and BoNT/A2 utilize three isoforms of synaptic vesicle protein SV2 (SV2A, B, and C) as their protein receptors. We here present a high resolution (2.0 Å) co-crystal structure of the BoNT/A2 receptor-binding domain in complex with the human SV2C luminal domain. The structure is similar to previously reported BoNT/A-SV2C complexes, but a shift of the receptor-binding segment in BoNT/A2 rotates SV2C in two dimensions giving insight into the dynamic behavior of the interaction. Small differences in key residues at the binding interface may influence the binding to different SV2 isoforms, which may contribute to the differences between BoNT/A1 and BoNT/A2 observed in the clinic.

## 1. Introduction

Botulinum neurotoxins (BoNTs) are among the most toxic substances known. They are produced by diverse anaerobic bacterium mainly classified as *Clostridium botulinum*. BoNTs block the release of neurotransmitters from peripheral nerve cells and cause botulism, a deadly paralysis requiring respiratory assistance. There are seven major serotypes of BoNT (denoted A–G), as well as numerous subtypes and mosaic variants [1]. A new serotype has been also recently reported, designated BoNT/X [2].

BoNTs are produced as a 150 kDa polypeptide chain that is post-translationally cleaved into two fragments, the N-terminal light chain (LC) of 50 kDa and the C-terminal heavy chain (HC) of 100 kDa. A disulfide bridge links HC and LC, and a loop from the HC wraps around the LC. The mature toxin has three domains [3]. The LC is a Zn^2+^-metalloprotease cleaving the intracellular target, SNARE proteins (soluble N-ethylmaleimide sensitive factor attachment protein receptor) thus preventing synaptic vesicle fusion and release of neurotransmitters. HC consists of a translocation domain (H_N_) and a receptor-binding domain (H_C_). H_N_ transports the LC across the cellular membrane [4], while H_C_ is responsible for anchoring the toxin to the neuronal cell surface, by binding to both a ganglioside [5] and a protein receptor. BoNT targets peripheral motorneurons, but has recently been shown to also undergo retrograde transport to the central nervous system [6]. BoNT binding to neurons is a sequential event where it first binds a ganglioside, enriching the toxin at the neuronal membrane, and then binds the protein receptor with high affinity and specificity [5,7,8,9,10,11,12,13,14,15,16,17,18,19]. BoNT/B, BoNT/G and BoNT/DC utilize synaptotagmin I and II as their protein receptors [8,9,13,15,18,19], while BoNT/A, BoNT/D, BoNT/E and BoNT/F instead use synaptic vesicle protein 2 (SV2) [7,10,11,12,14,16,17]. SV2 has three isoforms: SV2A, SV2B and SV2C, and the interacting part of the protein has been mapped to the fourth luminal domain [7,11,14]. Previously the structure of the receptor binding domain of BoNT/A1, a subtype of BoNT/A, in complex with the fourth luminal domain of human SV2C (SV2C-L4) [7] as well as its glycosylated form [20] has been reported [7,20].

There are several subtypes of BoNT/A (1–8), with amino acid sequence differences varying from 2.9% to 15.6% [1,21,22]. The Hc is usually the region where the majority of sequence variations occur. For instance, the Hc of BoNT/A2 (HcA2) displays 13% of amino acid sequence differences from HcA1. These variations are not likely to alter the receptors, but may have a significant impact on receptor binding properties, which in turn may impact the therapeutic efficacy [23]. BoNT/A1 is the first BoNT utilized in clinic and it remains the only BoNT/A for medical uses. However, there is a growing interest in exploring the medical use of BoNT/A2. It has been found that in an electrophysiological study in healthy individuals, BoNT/A2 is comparable to BoNT/A1 in terms of onset and duration of action [24]. Importantly, clinical trials for BoNT/A2 (A2NTX) (clinicaltrials.gov ID: NCT01910363) showed higher efficacy, earlier onset of action and better functional outcome with less spreading of its action than BoNT/A1 [25]. To understand how residue differences within the receptor binding domain of BoNT/A1 versus BoNT/A2 may impact their receptor recognition properties, we solved a high resolution crystal structure of HcA2 in complex with human SV2C-L4. Our structure is largely the same as the recently reported crystal structure of HcA2 + SV2C-L4 complex [26], but our structure offers a higher resolution (2.0 Å compared to 2.3 Å in the previous report), and revealed a different position of SV2C-L4, resulting from a significant rotation and shift of SV2C-L4.

## 2. Results and Discussion

### 2.1. Overall Structure of HcA2 and SV2C

HcA2 (BoNT/A2 residues 874 to 1296) was cloned from genomic DNA of *C. botulinum* Kyoto-F and expressed as a His6-tagged protein in *E. coli*. Human SV2C-L4 (residues 474 to 567) was expressed as His6-tagged protein also in *E. coli*. Following purification, the proteins were mixed in equimolar concentration and crystallized using the vapor diffusion technique.

**Figure 1:**
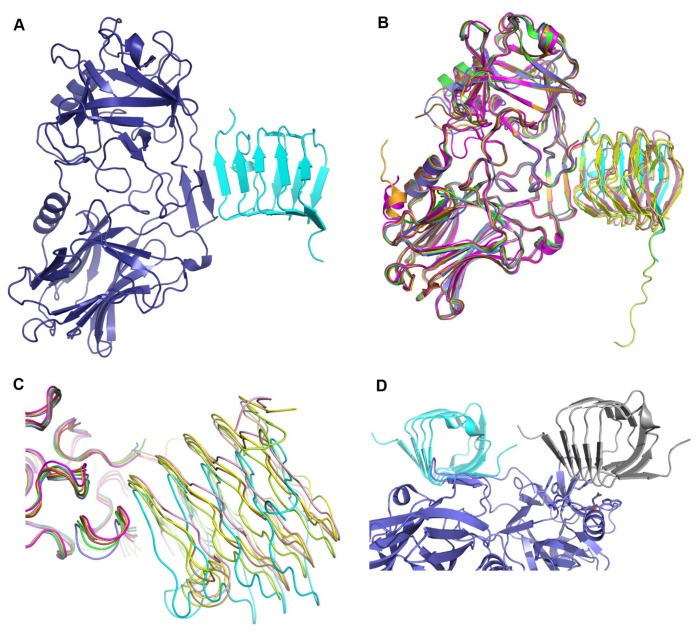
Overall interaction of the binding domain of botulinum neurotoxin (BoNT) with the human receptor SV2C. (**A**) Structure of BoNT binding domain A2 (HcA2) in complex with the human receptor SV2C-L4 (6ES1). Shown in cartoon with HcA2 in purple and SV2C-L4 in cyan, respectively. (**B**) Overlay of all HcA + SV2C-L4 structures aligned on HcA2 from 6ES1. Structures are 4JRA (HcA1 (Chain B) in brown and human SV2C (Chain C) in light brown), 5JLV (HcA1 (Chain B) in magenta and rat SV2C (Chain D) in pink), 5JMC (HcA1 (Chain A) in orange and glycosylated human SV2C (Chain B) in yellow), 5MOY (HcA2 (Chain A) in green and human SV2C (Chain B) in light green) and 6ES1 (HcA2 (Chain A) in blue and human SV2C (Chain B) in cyan). (**C**) Same overlay and colors as in (**B**) but shown with ribbon representation, zoomed in to the interaction site (β-hairpin) of HcA and SV2C-L4. (**D**) Crystal contact between the N-terminus of SV2C-L4 and the ganglioside-binding site of HcA2. HcA2 is shown in blue and human SV2C-L4 is shown in cyan. The symmetry related SV2C-L4 from a neighboring molecule is shown in grey. The residues involved in ganglioside-binding according to Stenmark et al. [5] are shown as sticks.

Relevant statistics on data collection and refinement for the crystal structure are shown in Table 1. The final protein model contains one molecule of HcA2, residues 876–1295, and one molecule of SV2C-L4, residues 474–567. The crystal structure has been reported to the PDB with accession code 6ES1. As shown in Figure 1A, HcA2 is highly similar in fold to the previously published HcA1 structure (3FUO) with RMSD of 0.65 Å over 409 Cα [12] and to the previously published structure of HcA2 (5MOY) with RMSD of 0.72 Å over 408 Cα [26]. HcA2 has two subdomains linked by a flexible region. The C-terminus of the right-handed quadrilateral β-helix of SV2C-L4 is bound to HcA2 by forming a continuation of the β-hairpin in HcA2 (residues 1141–1151), with an interaction surface area of 474 Å^2^ (Figure 1A). The SV2C-L4 structure is highly similar to the SV2C-L4 in complex with HcA1 found in 4JRA (Chain D) with RMSD of 0.74 Å over 92 Cα [7], the SV2C-L4 in complex with HcA2 found in 5MOY (Chain B) with RMSD of 0.44 Å over 94 Cα [26], and to the glycosylated SV2C-L4 in complex with HcA1 found in 5JLV (Chain D) with RMSD of 0.53 Å over 90 Cα [20]. Otherwise the most similar structures of SV2C found in PDB are the pentapeptide repeat proteins Qnrb1 from *Klebsiella pneumoniae* and Mfpa from *Mycobacterium tuberculosis* with RMSD of 1.69 over 82 Cα and 1.30 Å over 73 Cα, respectively [27,28].

### 2.2. Comparison to Reported HcA2-SV2C-L4 Structure

All previous structures of HcA1 or HcA2 in complex with SV2C-L4 (4JRA, 5JMC, 5JLV and 5MOY) when aligned (as shown in Figure 1B,C) overlap well for HcA and SV2C, as well as presenting similar mode of interactions. In the structure of HcA2 and SV2C presented herein (6ES1), SV2C-L4 has a different position with a rotation angle of up to 15° in the interaction site with HcA2, compared to what was observed previously (Figure 1B,C and Table 2). Also the interaction site β-hairpin of HcA2 in 6ES1 has moved up to 1.8 Å compared to all the other structures (Figure 1C).

**Figure 2:**
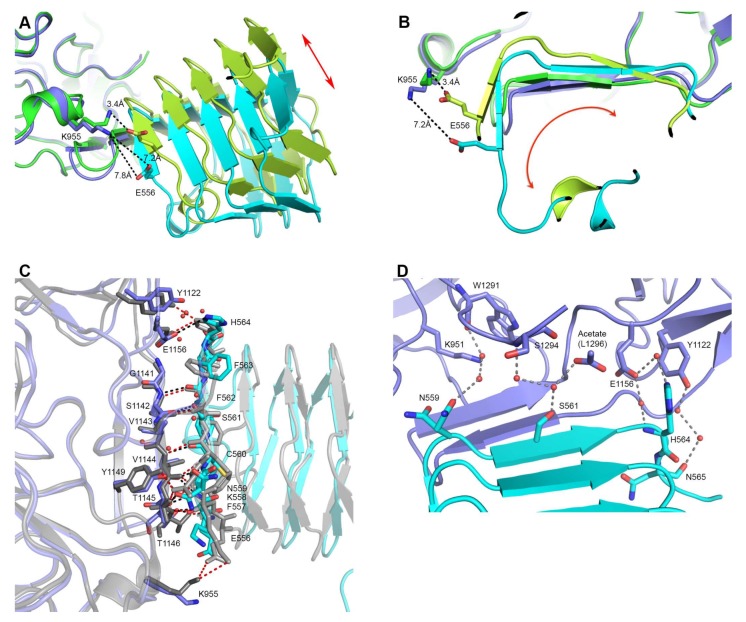
Interaction site differences between 5MOY and 6ES1. (**A**,**B**) Overlay of 6ES1 (HcA2 in blue and human SV2C-L4 in cyan) and 5MOY (HcA2 in green and human SV2C-L4 in light green). HcA2-K955 and SV2C-L4-E556 is shown in sticks. (**A**) Side view of the interaction site. Red arrow indicates domain shift. (**B**) Front view from SV2C-L4 perspective of the interaction site. Red arrow indicates domain rotation. (**C**) Detailed interaction site. Overlay of 6ES1 (HcA2 in blue and SV2C-L4 in cyan) and 5MOY (HcA2 in grey and SV2C-L4 in light grey). Important residues are shown as sticks and bridging water molecules of 6ES1 are shown as red spheres. Interactions of 6ES1 are shown as black dotted lines while interactions of 5MOY are shown as red dotted lines. (**D**) Bridging water interactions of 6ES1 are shown as grey dotted lines. Water molecules are shown as red spheres. HcA2 of 6ES1 is shown in blue and SV2C-L4 in cyan. Acetate molecule modeling the C-terminus of L1296 is shown as sticks.

When our new structure is overlaid with previously reported structure of the HcA2 + SV2C-L4 complex (5MOY), it is clear that SV2C-L4 has rotated in two directions. The part of the last (C-terminal) β-helical turn not interacting directly with HcA2 in 5MOY has moved closer to HcA2 as seen here in Figure 2A. Also the shift of the HcA2 β-hairpin causes SV2C-L4 to rotate in order to maintain the distances needed for the tight interactions of the continuing β-sheet (Figure 2B). A collection of the shifts between Cα-atoms of the HcA2 β-hairpin, SV2C-L4 interaction site and C-terminal β-helical turn, and the N-terminal β-helical turn are shown in Table 2. The HcA2 β-hairpin moves up to 1.8 Å, while the C-terminus of SV2C-L4 moves between 0.4–5.9 Å. However the residues of the interaction site of SV2C-L4 are only shifted by a maximum of 3 Å. The N-terminal segment of SV2C-L4 experiences much larger shift, between 3.0–8.1 Å as seen in Table 2. F562 of SV2C moves only 0.4 Å and is closest to the rotations turning point (Figure 2B). For SV2C-L4, the angle of the rotation was also calculated for the residues in Table 2. The reference point was then Cα of F562 in 6ES1. The shift is not uniform as it is composed of two rotations as previously described (Figure 2A,B).

All previous structures of HcA1 or HcA2 in complex with SV2C-L4 (4JRA, 5JMC, 5JLV and 5MOY) have different space groups and cell parameters from each other as summarized in Table 3, but upon aligning their structures (as shown in Figure 1B,C) they present very similar features, including the interaction between the toxin and its receptor. The structure of HcA2 and SV2C presented herein (6ES1) was solved in a different space group (see Table 1 and Table 3), which allowed us to observe a level of flexibility in the angle of interaction between SV2C and HcA2 (Figure 1B,C). This reveals a hinge-like flexibility in the BoNT/A-SV2C-L4 interaction. The crystallization pH may play a role in this shift. However, the pH for the crystallization of the structure presented here is 6.0, slightly less acidic than in 5JLV, which was solved at pH 4.6. Additionally, both 5MOY and 4JRA were crystallized at pH 7.5 (Table 3). It is however possible that construct sizes and residual purification tags could influence the crystal packing and thus the interaction of HcA and SV2C. A summary of the different construct sizes and purification tag cleavage is shown in Table 4.

In the crystal, the N-terminus of SV2C-L4 forms a crystal contact and packs against the previously determined ganglioside-binding site of the neighboring HcA2 molecule in the crystal (Figure 1D). This crystal contact is most likely the reason that Sialyl-T (ganglioside headgroup) [29], present in the crystallization condition, was not bound. Thus this crystal form prevents simultaneous binding of ganglioside and protein receptor to HcA2 [5]. This crystal contact may also be one of the reasons for the observed shift of SV2C-L4 in the crystal structure. 4JRA and 5MOY showed comparable interactions where the N-terminus of SV2C-L4 packs against the ganglioside-binding site of the neighboring HcA symmetry-related molecule; however, the crystal contacts differed between the structures.

Although many of the interactions are shared between the structure presented here and 5MOY (Figure 2C), noticeable variations are also observed. His564 of SV2C-L4 no longer makes electrostatic interactions to Tyr1122 of HcA2 in our structure as seen in Figure 2C. Also the rotation of SV2C-L4 causes the distance between Lys955 of HcA2 and Glu556 of SV2C-L4 to go from 3.4 Å to 7.2 Å and thus preventing formation of a salt bridge (Figure 2A,B). Additionally, eight water molecules are making several bridges between HcA2 and SV2C in the new structure, as shown in Figure 2D. These bridging waters are not observed in 5MOY, perhaps because of the lower resolution.

### 2.3. Comparison to Reported HcA1-SV2C-L4 Structure

**Figure 3:**
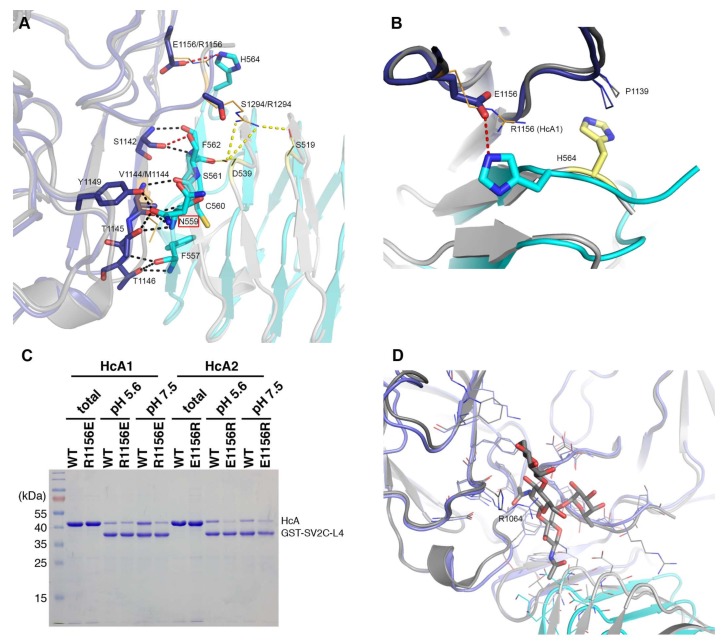
Detailed interactions between the BoNT binding domains and human SV2C-L4. (**A**) Polar interactions and salt bridges in the interaction interface of HcA2 and SV2C-L4 (6ES1) and HcA1 with SV2C-L4 (Chain B and C from 4JRA) are shown. For clarity, when no differences between HcA2 and HcA1 binding to SV2C-L4 are seen, only residues from HcA2 are shown. The glycosylation site at Asn559 is marked with a red box around the name. (**B**) Differences in the interaction of His564 in structures of HcA2 and human SV2C-L4 (6ES1) and HcA1 with human SV2C-L4 (Chain A and D from 4JRA). For both A and B: HcA2 is shown in blue, human SV2C-L4 from 6ES1 in cyan, HcA1 in dark grey and human SV2C-L4 from 4JRA in light grey. Residues from HcA1 and SV2C from 4JRA are shown as orange and yellow lines, respectively. Black dotted lines show interactions found for both HcA1 and HcA2, while red dotted lines show interactions only found for HcA2, and yellow dotted lines show interactions only found for HcA1. For the residues that differ from HcA2 to HcA1 within the interface, both residue names are shown, with HcA1 annotation last. (**C**) GST or GST-fused human SV2C-L4 were immobilized on beads and incubated with WT or mutant HcA1 and HcA2. Bound materials were analyzed by SDS-PAGE and Coomassie staining. (**D**) Overlay of interaction interface of HcA2 and SV2C-L4 (6ES1) and HcA1 with SV2C-L4 (Chain A and C from 5JLV). Glycosylation and residues within 6 Å of glycosylation of N559 are shown in sticks and lines, respectively. HcA2 is shown in blue, SV2C-L4 from 6ES1 in cyan, HcA1 in dark grey and SV2C-L4 from 5JLV in light grey.

An overlay of HcA2 and HcA1 in complex with SV2C-L4 (6ES1 and Chain B and C from 4JRA) is shown in Figure 1B. As expected, HcA1 and HcA2 are highly similar with an overall RMSD of 0.82 Å over 406 Cα (Chain B of 4JRA) [7]. As previously shown, SV2C-L4 displays a significant shift of the N-terminal part between the two structures, but the interactions between the C-terminal part of SV2C-L4 and HcA remains similar for the two subtypes and is mostly built up from hydrogen bonds to the backbone of SV2C (Figure 3A).

Ser561 of SV2C-L4 forms one hydrogen bond from the side chain hydroxyl group to the backbone carbonyl of Ser1142 in HcA2, which is not found in the structure with HcA1 since Ser561 has a different conformation.

His564 from SV2C-L4 forms a salt bridge with Glu1156 of HcA2. In HcA1, position 1156 is an arginine, which could repel the histidine, and is also seen in 4JRA where His564 instead interacts with Pro1139 (Figure 3B) [7]. As shown in Figure 3C, a mutation of Glu1156 in HcA2 to arginine as in HcA1 lowers the binding to SV2C. This can be explained by the lost interaction to His564 of SV2C. A mutation of Arg1156 to glutamate in HcA1 also seem to lower the binding, although not as significantly as for the HcA2 mutation. Several mutations, including Arg1156 to glutamate have been shown to lower SV2C binding [7,20]. Interestingly, HcA1 seems to interact more strongly with SV2C at pH 7.5 than pH 5.6 (Figure 3C), suggesting that one or more interactions are pH-dependent.

**Figure 4:**
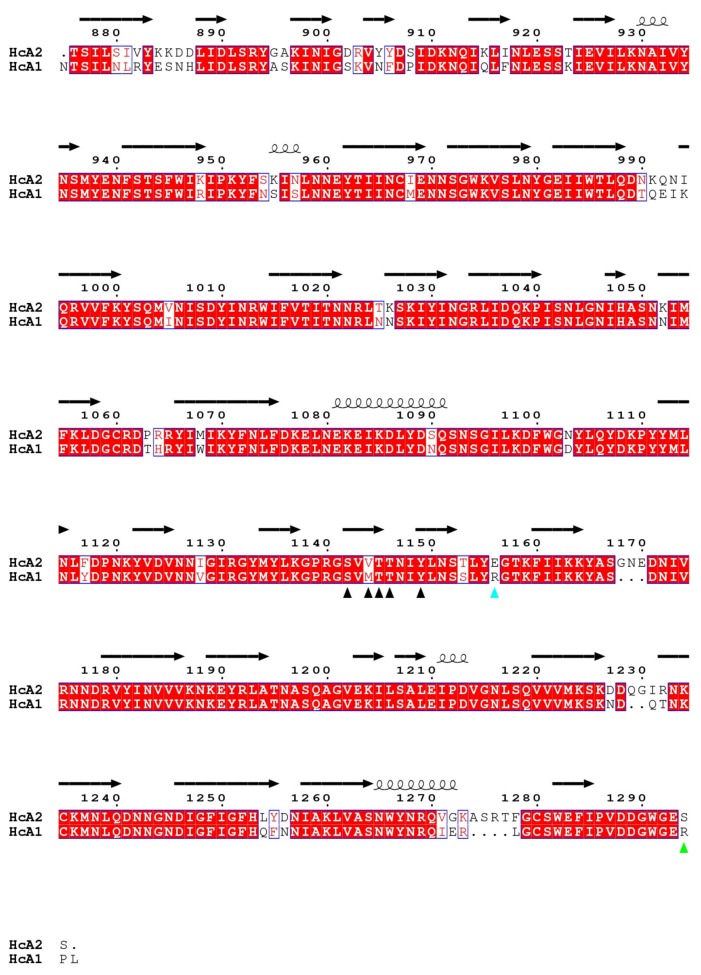
Structural sequence alignment of HcA1 and HcA2 from 4JRA (Chain B) and 6ES1. Red boxes mark conserved residues and white boxes weakly conserved residues. Residues that interact for both HcA1 and HcA2 are marked with a black arrowhead, while residues that only interact for either HcA1 or HcA2 are marked with green or cyan arrowheads, respectively.

HcA1 uses Arg1294 to form salt bridges and a hydrogen bond to Glu539 and Ser519 of SV2C-L4 respectively, interactions that HcA2 does not possess since Ser1294 in HcA2 cannot reach those interaction partners (Figure 3A). A mutation of Arg1294 to alanine in HcA1 was reported to significantly lower binding of SV2C [7]. A full structural sequence comparison between HcA2 from 6ES1 and HcA1 from 4JRA (Chain B) [7] can be found in Figure 4.

A mutation of Phe563 of SV2C-L4 to alanine almost abolishes the binding to HcA1 [7]. This is interesting since Phe563 sidechain has different conformations when comparing different structures of HcA and SV2C (Figure 2C) [7,20,26]. In the structure presented herein, it may interact with the C-terminal carboxylate of HcA2 (Leu1296), where only an acetate ion could be modeled due to the lack of electron density for the entire residue (Figure 2D).

### 2.4. SV2 Sequence Comparison

**Figure 5:**
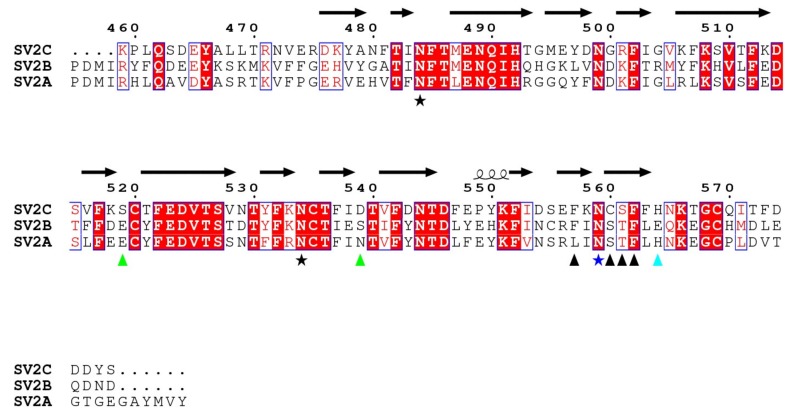
Sequence alignment of the fourth luminal domains from human SV2A, SV2B and SV2C. Red boxes mark conserved residues and white boxes weakly conserved residues. Residues of SV2C that interact with both HcA1 and HcA2 are marked with a black arrowhead or a blue star, while residues that only interact with either HcA1 or HcA2 are marked with green or cyan arrowheads, respectively. Conserved glycosylation sites are marked with stars, with the glycosylation involved in interaction in vivo [10,16] shown in dark blue. Secondary structure elements are from SV2C in 6ES1. Observe that in 6ES1, only residues 474–567 of SV2C are seen.

**Figure 6:**
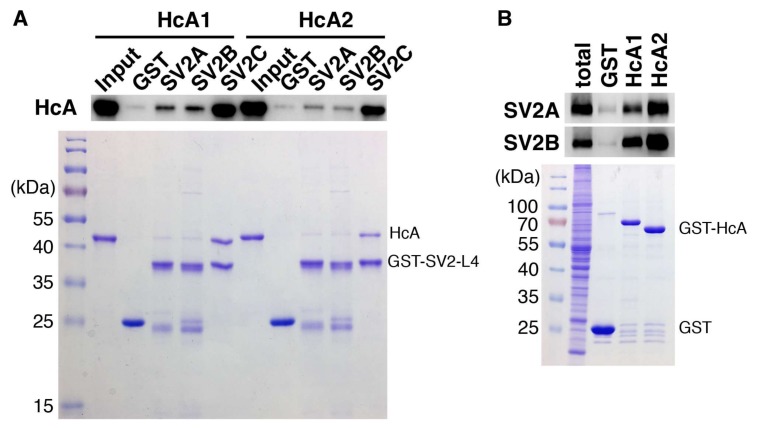
Binding of HcA1 and HcA2 to SV2A, SV2B and SV2C characterized by pull-down assays. (**A**) GST or GST-fused recombinant human SV2A-L4, human SV2B-L4 or human SV2C-L4 were immobilized on beads and then incubated with HcA1 and HcA2 in pull-down assays. Bound HcA1 and HcA2 were detected by immunoblot analysis using an HA antibody that recognizes the HA tag in HcA1 and HcA2 (upper panel). The proteins immobilized on beads were analyzed by SDS-PAGE followed by visualization by Coomassie staining to ensure equal loading of bait proteins (lower panel). (**B**) GST or GST-fused receptor binding domains of BoNT/A isoforms were incubated with rat brain detergent extracts. Bound materials were analyzed by immunoblot detecting SV2A and SV2B using their specific polyclonal antibodies (upper panel). The proteins immobilized on beads were analyzed by SDS-PAGE followed by visualization by Coomassie staining to ensure equal loading of bait proteins (lower panel).

A sequence comparison of the fourth luminal domains of human SV2A, SV2B and SV2C is shown in Figure 5, with residues of SV2C interacting with HcA1 and HcA2 marked. Residues in positions 519 and 539 of SV2C that interact with Arg1294 in HcA1 are not conserved in SV2A and SV2B. However, similar pairs of polar or negative residues, with Ser-Asp, Glu-Ser and Glu-Asn, are found in position 519 and 539 for SV2C, SV2B and SV2A, respectively. All are therefore able to interact with the positively charged Arg1294 of HcA1. Likely the change of Arg1294 to serine in HcA2 is at least partly responsible for the lower degree of binding of HcA2 to non-glycosylated SV2A and SV2B seen in Figure 6A. His564 in SV2C that interacts with Glu1156 of HcA2 is conserved in SV2A but not in SV2B where instead a negatively charged Glu is found, which would repel the Glu1156 in HcA2 (Figure 3B and Figure 5). This is consistent with HcA2 showing weaker binding to non-glycosylated SV2B than to SV2A and SV2C (Figure 6A).

Of the three conserved glycosylation sites, the one closer to the C-terminus, Asn559 in SV2C, is found in the interaction interface (Figure 3A and Figure 5) and was shown to be critical for binding of BoNT/A [10,16]. Previous study showed that a mutation of Asn559 to alanine does not influence SV2C binding to HcA1 significantly [7]. The specific hydrogen bond to the side chain of HcA would appear to not contribute significantly. Glycosylation of Asn559 has been found to strengthen BoNT/A binding [10,16,20,30]. In HcA2, this amino acid is modeled in two positions and thus the side chain is flexible and could accommodate a glycan. Our structure suggests that only Arg1064 in HcA2 would need to change conformation for this glycan to be able to bind HcA2 in a similar manner to HcA1 (Figure 3D) [20].

HcA2 has a hydrogen bond to the hydroxyl group of Ser561 on SV2C. That position is instead a threonine in SV2A and SV2B, and thus may still interact with HcA2; however, this change could also lower the affinity. In chain B and C of 4JRA [7], the corresponding atoms of Ser1142 in HcA1 and Ser561 in SV2C-L4 are only 4.5 Å apart, and may form a hydrogen bond upon conformational change of Ser561.

### 2.5. In Vitro Binding Assay

The binding of HcA1 and HcA2 to different non-glycosylated SV2 isoforms was determined and the results are presented in Figure 6A. HcA1 shows binding to all three isoforms of SV2, while HcA2 binds only to SV2C strongly. This finding is consistent with the change of Arg1294 and Arg1156 in HcA1 to serine and glutamic acid in HcA2, respectively, as pointed out previously. The change of Ser561 in SV2C to threonine in SV2A and SV2B may also affect the binding. When instead testing the ability of HcA1 and HcA2 to bind the glycosylated SV2A and SV2B by pull-down of rat brain extract, both HcA1 and HcA2 bind SV2A and SV2B, suggesting that the presence of glycan can compensate any reduction in protein–protein interactions for HcA2-SV2C-L4 complex (Figure 6B). The binding to SV2C was further tested by pull-down using recombinant proteins (Figure 3C). Interestingly, HcA1 binds SV2C stronger at pH 7.5 than at pH 5.6 while HcA2 appear to bind SV2C slightly less at pH 7.5 compared to pH 5.6 (Figure 3C). These results suggest that HcA2 may hold onto SV2 tighter than HcA1 within the acidic environment of endosomes, which may influence the translocation process and reduce retrograde transport.

The differences in the interactions with the various SV2 isoforms can be one of the reasons for the differences between BoNT/A1 and BoNT/A2 observed in the clinic. Subtle differences in receptor interactions could contribute to the onset, duration and effect of the BoNTs. Detailed studies of the receptor interactions are an important part of understanding, predicting and evaluating clinical effects.

## 3. Materials and Methods 

Cloning, expression and purification of HcA2. Clostridium Botulinum A2 Kyoto-F Neurotoxin Binding domain (HcA2, residues 874-1296 of Uniprot ID Q45894) was amplified from *C. Botulinum* A2 Kyoto-F genomic DNA (primers 5′-GCTGCTCATATGGTTAATACCTCTATATTGAGTATAGTATATAAAAAAGATG-3′ and 5′-GCTGCTCTCGAGTTACAGTGAACTTTCTCCCCATC-3′) and inserted using NdeI and XhoI restriction sites into a modified pET-28a vector (Novagen, Birmingham, UK) in which the thrombin site following the N-terminal His6-tag has been replaced by a TEV site. Correct construct was ensured using sequencing. HcA2 protein was expressed in *E. coli* BL21 (DE3) (Novagen) by induction with 0.5 mM IPTG at an OD_600_ of 1.5 in Terrific Broth media and further grown at 20 °C for 19 h. Bacteria were harvested by centrifugation and lysed in Lysis buffer (50 mM KPi, pH 7.5, 200 mM NaCl, 10% glycerol) after treatment with lysozyme, 5 mM MgSO_4_, DNAse and protease inhibitor cocktail (Roche, Basel, Switzerland) using high-pressure homogenization followed by centrifugation. His-tagged HcA2 was purified on gravity flow column (Econo-Pac Chromatography column, Bio-RAD, Hercules, CA, USA) after incubation with Ni-NTA (0.5 mL/50 mL cleared lysate) and 15 mM Imidazole, washed with Buffer A (50 mM KPi, pH 7.5, 200 mM NaCl, 50 mM Imidazole) followed by elution of the protein with Buffer B (50 mM Tris-HCl, pH 7.4, 300 mM NaCl, 500 mM Imidazole). HcA2 containing fractions were loaded on a Superdex 75 16/60 column (GE Healthcare, Uppsala, Sweden) and separated using Buffer C (20 mM Tris-HCl, pH 7.4, 300 mM NaCl, 10% glycerol). Fractions containing HcA2 were pooled and purity was analyzed on SDS-PAGE. Protein was concentrated using Vivaspin 20 (Sartorious Stedim, Goettingen, Germany) 30 kDa MWCO at 4800 rcf at 4 °C. Concentration was determined using a calculated extinction coefficient of 88,950 M^−1^s^−1^.

Cloning, expression and purification of SV2C-L4*.* The interacting part of the fourth luminal domain of synaptic vesicle glycoprotein 2C (SV2C-L4, residues 474-567 Uniprot ID Q496J9) was amplified from the pGEX4-T1 SV2C-L4 plasmid used for binding studies (primers 5′-TACTTCCAATCCATGGAGAGAGATAAATATGCAAATTTC-3′ and 5′-TATCCACCTTTACTGTCACGTCTTGTTGTGAAAAAACGAG-3′) and cloned into a pNIC28-Bsa4 (N-terminal His6-tag with TEV site, KanR) vector using LIC cloning. Correct construct was ensured using sequencing. SV2C-L4 protein was expressed in *E. coli* BL21 (DE3) T1R (SigmaAldrich B2935, St. Louis, MO, USA), that had been transformed with pRARE2 and made competent, by induction with 0.5 mM IPTG at an OD_600_ of 3.0 in Terrific Broth media and further growth at 18 °C overnight. Bacteria were harvested by centrifugation and lysed in Lysis buffer (100 mM HEPES, pH 8.0, 500 mM NaCl, 10% (*v*/*v*) glycerol, 10 mM Imidazole, 0.5 mM TCEP) after treatment with benzonase nuclease (Sigma) and protease inhibitor cocktail (Roche) using sonication followed by centrifugation. His-tagged SV2C-L4 was purified on 2 mL HisTrap HP (GE Healthcare), washed with Buffer D (20 mM HEPES, pH 7.5, 500 mM NaCl, 10% glycerol, 10 mM Imidazole, 0.5 mM TCEP) and then Buffer D with 50 mM Imidazole, followed by elution of the protein using Buffer D with 500 mM Imidazole. SV2C-L4 containing fractions were loaded on a Superdex 75 HiLoad 16/60 column (GE Healthcare) and separated using Buffer E (20 mM HEPES, pH 7.5, 300 mM NaCl, 10% glycerol, 0.5 mM TCEP). Fractions containing SV2C-L4 were pooled and its purity was analyzed on SDS-PAGE. The protein was concentrated using a centrifugal concentrator. The concentration was determined using a calculated extinction coefficient of 7450 M^−1^s^−1^. The protein size was verified with mass spectrometry.

Crystallization. HcA2 and SV2C-L4 were mixed at 160 μM each (8.27 and 2.24 mg/mL, respectively). Two millimolar Sialylated Thomsen–Friedenreich carbohydrate antigen (Sialyl-T, purchased from Carbosynth, UK) was added prior to crystallization and mixture was incubated in room temperature for 15 min. Sitting drop vapor diffusion at 20 °C was performed and protein mixture was mixed with reservoir solution (200 mM CaCl_2_, 100 mM MES pH 6.0, 20 % PEG 6000) in a 1:1 ratio. Diffraction quality crystals appeared within three weeks, and were subsequently frozen in liquid nitrogen with a mix of 40% glycerol and 50% reservoir solution as cryo-protectant. Data collection was performed at beamline 14.1 at BESSY, Germany, at 100 K using a wavelength of 0.919908 Å. Data reduction and processing was carried out using XDS [31,32] and programs from the CCP4 suite [33]. Relevant statistics can be found in Table 1.

The structure was solved via molecular replacement using Phaser [34] with residues 871–1295 from pdb 3BTA [35] as search model for HcA2 and chain C from pdb 4JRA [7] as search model for SV2C-L4. Refinement in Refmac5 [36,37], interspersed with manual building in Coot [38,39] was needed to complete the model. Water molecules were automatically placed in the maps, using a F_O_–F_C_ Fourier difference map cutoff of 3 σ, and subsequently validated to ensure correct positioning. Ramachandran statistics were generated using MolProbity [40,41]. The structure has been deposited in the protein data bank with accession code 6ES1. All structure figures were prepared using PyMOL (http://www.pymol.org).

Interaction analysis using PISA. Protein–protein interaction interface calculations for HcA2 with SV2C (6ES1) was generated using the ‘Protein interfaces, surfaces and assemblies’ service PISA at the European Bioinformatics Institute. (http://www.ebi.ac.uk/pdbe/prot_int/pistart.html) [42,43,44].

Structural sequence alignment. Structure based sequence alignment of HcA1 (Chain B from 4JRA) [7] and HcA2 (Chain A from 6ES1) binding to SV2C-L4 was generated using PDBeFOLD [45]. Graphic representation was generated using ESpript 3.0 [46] with the secondary structure from HcA2 (Chain A from 6ES1).

Sequence alignment. Sequence alignment of the fourth luminal domains of SV2A (residues 469–598 from Uniprot ID Q7L0J3), SV2B (residues 412–535 from Uniprot ID Q7L1I2) and SV2C (residues 459–578 from Uniprot ID Q496J9) was performed using Clustal Omega v 1.2.1 (2014, Dublin, Ireland) [47]. Graphic representation was generated using ESpript 3.0 (2013, Lyon cedex, France) [46] with the secondary structure from SV2C-L4 (Chain B from 6ES1).

Cloning and production of proteins for binding studies. The L4 domain of human SV2A (residues 468–596 from Uniprot ID Q7L0J3), SV2B (residues 410–536 from Uniprot ID Q7L1I2) and SV2C (residues 454–580 from Uniprot Q496J9) were cloned into pGEX4-T1 from human cDNA purchased from Harvard PlasmID Database. The H_C_A1 (residues 876–1296 from Uniprot ID A2I2U2) and H_C_A2 (residues 874–1296 of Uniprot ID Q45894) were cloned into pGEX4-T1 as GST fusion proteins, and into pET28a with a HA tag fused to the N-terminal site as His6-tagged proteins. Proteins were expressed in BL21 (DE3). The bacteria were firstly cultured at 37 °C. When OD_595_ reached to 0.8, it was induced by 0.1 mM IPTG at 22 °C overnight. Bacteria were harvested and lysed with lysis buffer (50 mM Tris pH 7.5, 150 mM NaCl and protease inhibitor). After centrifugation, the supernatants were loaded onto GSTrap FF column (GE Healthcare). Proteins were eluted with 10 mM reduced Glutathione in lysis buffer. For His6-tagged H_C_A1 and HcA2, supernatants were loaded onto Histrap FF column (GE Healthcare) and proteins were eluted with gradient imidazole (0–500 mM) in lysis buffer. After elution, proteins were concentrated into 2 mL volume and desalted using PD-10 column (GE Healthcare) to remove reduced glutathione and imidazole. Purified proteins were stored at −80 °C. The mutants (HcA1 R1156E and HcA2 E1156R) were generated by site-directed mutagenesis and verified by sequencing.

Preparation of rat brain extract. Sprague Dawley (CD IGS) rat was purchased from Charles River Laboratories (Cambridge, MA, USA). Rat brain was homogenized in 15 mL 320 mM sucrose buffer, followed by centrifugation at 5000 rpm for 2 min at 4 °C. Supernatants were collected and centrifuged at 11,000 rpm for 12 min. The pellet was collected and solubilized in 15 mL Tris buffer (TBS: 20 mM Tris, 150 mM NaCl) with 2% of Triton X-100 and a cocktail of protease inhibitors (Roche, Indianapolis, IN, USA). Samples were centrifuged at 17,000 rpm for 20 min to remove insoluble materials. The final brain detergent extract concentration was ~2 mg/mL.

HcA pull-down by rat brain extract. Thirty microliter GST (Glutathione) beads were incubated with 20 µg GST or GST-fused receptor binding domains of BoNT/A isoforms in Hepes buffer (50 mM Hepes pH 7.5, 150 mM NaCl). After incubation for 30 min at 4 °C, the bead baits were washed three times with same buffer. Then beads baits were incubated with 500 µL rat brain extract for 1 h at 4 °C. After incubation, beads were washed three times again with same buffer, then 40 µL 1× SDS loading buffer was added and the samples were heated at 65 °C for 5 min. Five microliters of each sample was loaded into 12.5% SDS-PAGE for analysis and followed by western blot. Nitrocellulose membranes were blot with polyclonal SV2A antibody (Synaptic System, catalog #119002) and SV2B antibody (Synaptic System, catalog # 119102).

SV2 binding with HcA1 and HcA2. Thirty microliter GST (Glutathione) beads were incubated with 15 µg GST or GST-fused human SV2C-L4 in Hepes buffer (50 mM Hepes pH 7.5, 150 mM NaCl) or in MES buffer (50 mM MES pH 5.6, 150 mM NaCl). After incubation for 30 min at 4 °C, the bead baits were washed three times with same buffer. Then beads baits were incubated with 20 µg HcA1 or HcA2 wt and mutants in 200 µL corresponding buffers for 1 h at 4 °C. After incubation, beads were washed three times again with same buffer, then 50 µL 1× SDS loading buffer was added and the samples were boiled for 5 min. Five microliters of each sample was loaded into 12.5% SDS-PAGE for analysis.

## Figures and Tables

**Table 1:** Crystallographic data collection and refinement statistics.

Data Collection	HcA2 + SV2C-L4 Complex
Space group	P2_1_2_1_2
Cell dimensions	
*a*, *b*, *c* (Å)	100.7, 122.1, 47.7
*α*, *β*, *γ* (°)	90, 90, 90
Resolution (Å)	47.66–2.00 (2.05–2.00)
R_merge_ (%)	24.2 (123.7)
I/σ (I)	9.4 (2.2)
Completeness (%)	99.8 (97.7)
CC (1/2) (%)	99.6 (48.1)
Redundancy	13.2 (13.4)
**Refinement**	
Resolution (Å)	47.66–2.00
No. unique reflections	40,692 (2897)
R_work_/R_free_	18.14/22.97
Wilson B factor (Å^2^)	24.7
No. atoms	
HcA2	3471
SV2C-L4	813
Water	359
Acetate	4
B-factors (Å^2^)	
HcA2	27.9
SV2C-L4	27.2
Water	36.2
Acetate	39.9
RMS deviations	
Bond lengths (Å)	0.0147
Bond angles (°)	1.6628
Ramachandran plot, residues in (%)	
Most favourable region	96.09
Additional allowed region	3.91

Highest resolution shell is shown in parenthesis.

**Table 2:** Distances between Cα-atoms of residues from 5MOY and 6ES1 when overlaid based on HcA2.

Protein	Residue	Distance (Å)	Angle (°) *
HcA2	G1141	0.5	
	S1142	0.5	
	V1143	0.3	
	V1144	0.7	
	T1145	1.2	
	T1146	1.8	
	N1147	1.8	
	I1148	0.9	
	Y1149	0.5	
	L1150	0.5	
SV2C-L4	E556	3.9	15.6
	F557	2.4	12.4
	K558	2.3	10.6
	N559	1.8	9.6
	C560	1.4	11.2
	S561	0.9	12.1
	F562	0.4	Not applicable
	F563	1.1	16.1
	H564	2.6	21.3
	N565	2.8	22.8
	K566	2.6	13.6
	D546	3.0	16.8
	F547	3.0	18.7
	E548	4.0	22.4
	P549	4.5	23.1
	Y550	5.5	21.5
	K551	4.9	20.1
	F552	4.7	19.6
	I553	5.9	19.8
	D554	5.9	18.5
	S555	4.7	17.4
	N480	6.6	18.0
	F481	6.1	17.0
	T482	4.8	14.4
	I483	4.5	13.6
	N484	3.0	8.8
	F485	4.3	11.2
	T486	4.4	11.8
	M487	5.7	14.7
	E488	6.3	15.4
	N489	7.4	16.8
	Q490	7.7	17.9
	I491	7.8	18.7
	H492	7.3	18.4
	T493	8.0	19.1
	G494	8.1	19.3
	M495	7.2	18.6
	E496	6.5	18.3
	Y497	5.9	18.4

* Determined by taking the angle between the Cα-atoms compared to 6ES1/SV2C-L4/F562-Cα.

**Table 3:** Crystal space group, cell parameters and pH of crystallizations and protein buffers.

Structure	Reference	Space Group	Cell Parameters *a*, *b*, *c* (Å)	Cell Parameters *α*, *β*, *γ* (°)	HcA Buffer pH	SV2C Buffer pH	Crystal Condition pH
6ES1	This work	P2_1_2_1_2	100.7, 122.1, 47.7	90.0, 90.0, 90.0	7.4	7.5	6.0
5MOY	[26]	P2_1_2_1_2_1_	47.7, 91.6, 147.7	90.0, 90.0, 90.0	7.5	7.5	7.5
5JMC	[20]	P12_1_1	88.7, 144.0, 110.9	90.0, 93.6, 90.0	7.5	7.5	6.5
5JLV	[20]	C121	109.0, 111.9, 126.3	90.0, 101.3, 90.0	7.5	7.5	4.6
4JRA	[7]	C121 *	115.4, 105.3, 128.0	90.0, 90.02, 90.0	**	**	7.5

* (twin operator -h, -k, l); ** Not stated.

**Table 4:** HcA and SV2C construct sizes and purification tag cleavage.

Structure	HcA Construct (Start → End)	HcA His-Tag Cleaved?	SV2C-L4 Construct (Start → End)	SV2C-L4 His-Tag Cleaved?
6ES1	874–1296	No	474–567	No
5MOY	871–1296	Yes	456–574	No
5JMC	872–1296	Yes	455–577 (rat)	Yes
5JLV	872–1296	Yes	473–567	Yes
4JRA	871–1296	No	456–574	No
